# Evaluating Potential for Using Municipal Sewage Sludge in the Rehabilitation of Ground Degraded by the Sodium Processing Industry

**DOI:** 10.1007/s00128-018-2517-z

**Published:** 2018-12-11

**Authors:** Alicja Kicińska, Jarosław Gucwa, Beata Kosa-Burda

**Affiliations:** Faculty of Geology, Geophysics and Environmental Protection, Department of Environmental Protection, AGH University of Science and Technology, Mickiewicza 30 av, 30-059 Krakow, Poland

**Keywords:** Municipal sewage sludge, Landfill, Subsoil, Rehabilitation

## Abstract

The paper attempts at evaluating potential for the use of sewage sludge produced in a rural area for the rehabilitation of ground degraded by the sodium processing industry. The results demonstrated that the studied sewage sludge conforms to the current regulatory standards, enabling its use in land rehabilitation, both for agricultural and non-agricultural purposes. Ground in areas degraded by the KZS “Solvay” industrial plant has poor parameters in terms of pH, and moderate parameters in terms of humic substance content. An experiment using the bearded iris (*Iris barbata*) demonstrated that sewage sludge from the “Stróże” treatment plant stimulates plant growth. Therefore, it can be used both for ground rehabilitation, and for fertilization. Considering its properties and its broad spectrum of potential uses, sewage sludge should be viewed as a resource rather than a waste product.

The management of sewage sludge, the by-product of municipal sewage treatment, is one of the greatest challenges in modern waste management. Its potential utilization for agricultural, non-agricultural, and soil rehabilitation purposes is contingent upon the identification of its physical, chemical, and microbiological properties (Cavalheiro et al. 2017; Kępka et al. 2017; Klojzy-Karczmarczyk and Mazurek 2017). It seems impossible for sewage sludge production to be limited, and it should rather be expected to increase instead. This is mainly associated with the considerable development of sewer systems, particularly in rural areas. The problem of sewage sludge management lies primarily in the complex composition of the material, being a mixture of solids, liquids, and dissolved gases. Such a composition necessitates complex treatment processes that are neither fully controllable, nor fully predictable, while the maintenance of stable conditions for these processes requires a number of parameters to be monitored.

Improper management of sewage sludge generates the threat of toxic substances (e.g. heavy metals) leaching into other parts of the environment, e.g. soil, destroying soil fauna and flora (Kicińska 2019). Another risk is the potential subsoilwater contamination due to inadequate treatment, storage, or transport of sewage sludge. Legally (European Directive 86/278/EEC, the Polish Waste Act, 2012), sewage sludge can be used in agriculture for soil fertilization for the production of commercial food crops, animal feed, compost, and non-food crops, as well as for land rehabilitation for agricultural and non-agricultural purposes (Antonkiewicz 2010; Kicińska et al. 2018; Kicińska 2018).

The rehabilitation of degraded land may take place spontaneously through a variety of biochemical processes occurring in the environment. However, technical and biological land rehabilitation methods are commonly used, often simultaneously. Rehabilitation processes result in the preparation of the land for forest, agricultural, or commercial use. The latter typically involves using the land for recreation, services, or industrial purposes (Kicińska 2016a, b). Currently, land application is the dominant method of sewage sludge disposal. Sewage sludge can be applied in land rehabilitation to improve the physical, chemical, or biological properties of the soil. It can be applied in various forms, including liquid, solid, or semi-solid. However, sewage sludge cannot be applied in a way that would be harmful to the soil, and as a consequence, to the environment (Tarnawski and Baran 2018). In accordance with the Polish Regulation of the Minister of the Environment on municipal sewage sludge (2015), the applied doses of sewage sludge should not exceed: 3 Mg dw/ha/year in agricultural land or land rehabilitated for agricultural purposes, or 15 Mg dw/ha/year in land rehabilitated for non-agricultural purposes or in the case of other uses listed in the Waste Act (2012). The exact dosage is selected so as to avoid exceeding critical values for heavy metal content in the topsoil, up to a depth of 25 cm, after application. Land application of sewage sludge is restricted by the risk of toxic substance (e.g. heavy metal) penetration into the soil and water. The presence of any pathogenic organisms, adversely affecting soil hygiene, is another important factor.

With these considerations in mind, the present paper attempts at evaluating potential for the use of sewage sludge produced in a rural area for the rehabilitation of ground degraded by the sodium compound mining and processing industry. The objectives of the study were as follows:

1. to determine the basic parameters of sewage sludge and of ground degraded by the sodium processing industry.
2. to investigate whether the studied sewage sludge conforms to the applicable regulatory standards regarding its use for land rehabilitation.
3. to evaluate the growth of a bulbous plant (bearded iris) in two substrates: M1, a mixture of subsoil and sewage sludge, and M2, degraded subsoil, as a means of verifying the impact of the studied waste product on the nutrient parameters of subsoil.

## Materials and Methods

Two materials were subjected to chemical analysis: (1) sewage sludge from the “Stróże” sewage treatment plant (Grybów commune, southern Poland), and (2) subsoil degraded by the KZS “Solvay” sodium processing plant.

A sample of sewage sludge, comprising approx. 10 kg of an organic and mineral suspension, was collected in accordance with the PN-EN ISO 5667-13:2011 standard in March 2016. It was stored in a tightly sealed plastic bucket to keep air and light out. The studied sludge was black in color, an indication that it had been well digested, and had an unpleasant, moldy odor. It was morphologically homogeneous, lumpy, with a cellular structure. The sewage treatment plant where the sludge was produced uses a mechanical and biological treatment process, beginning with mechanical processing, followed by biological treatment, after which unstabilized sewage sludge undergoes thickening, sanitization, and neutralization.

Subsoil samples were obtained in May 2016, from brownfield land previously occupied by the KZS “Solvay” industrial plant. The KZS plant, established in 1906, produced sodium carbonate (Na_2_CO_3_) and lye (NaOH). Later, it expanded its production to include salmiac (NH_4_Cl) and food-grade carbon dioxide (CO_2_). In the early 1970s, the plant began to recover calcium chloride (CaCl_2_) from distillation waste products. The waste mainly comprised calcium sludges deposited in settling basins. The total weight of waste products deposited by the “Solvay” plant is estimated at approx. 5,000,000 Mg.

**Fig. 1:**
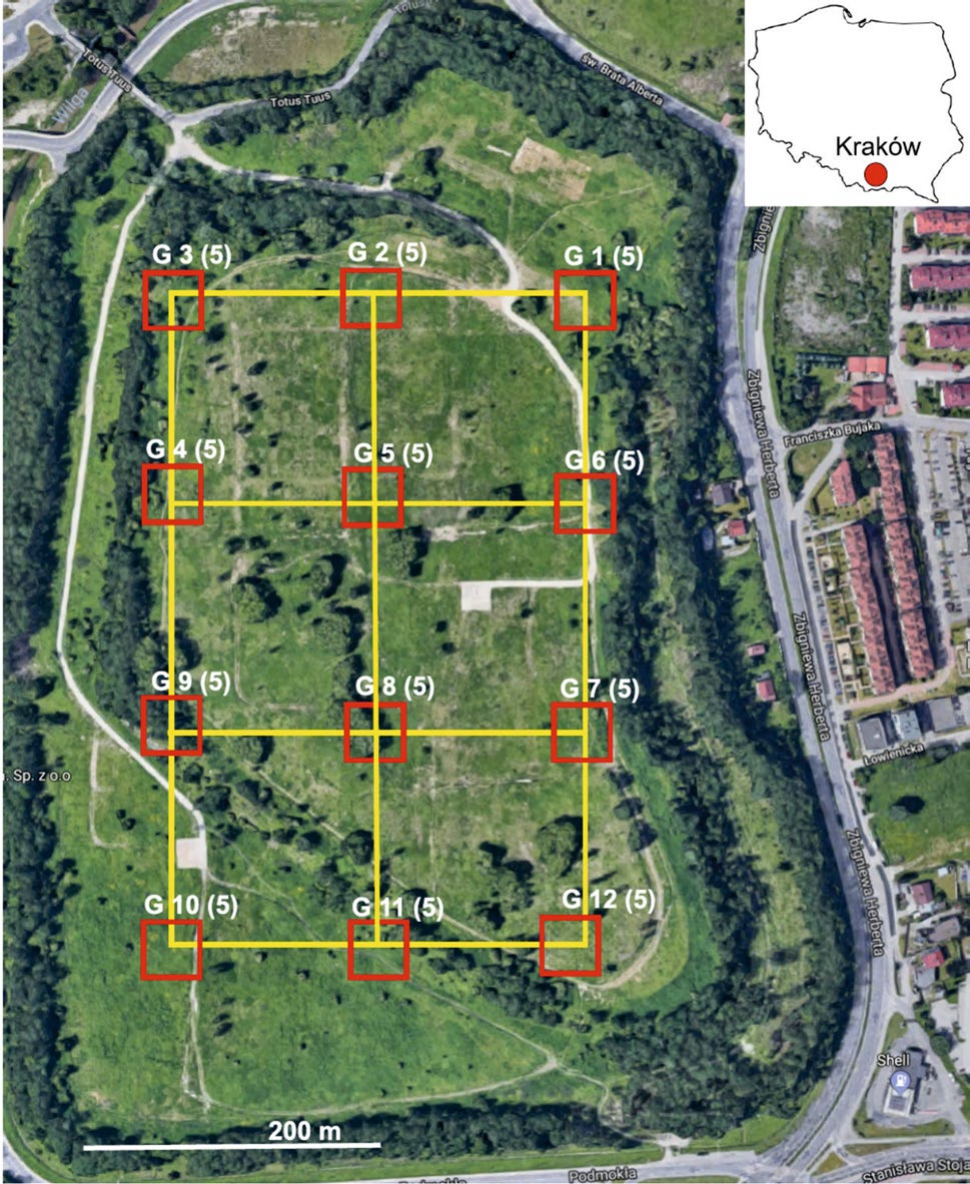
Sampling sites. (source: Google Earth); G (5)—number of sampling point (number of samples)

The subsoil sampling model developed in 2016 provided for the collection of samples from 12 sites, making up six square plots with a side length of approx. 100 m (Fig. 1). Two subsoil types, distinguished by color, dominated in the collected samples. One subsoil type was dark brown, the other—light gray. The dark brown subsoil samples contained spots of black-and-gray coloration, had a low moisture content, and a clay-like, compacted, hard structure. When crushed by hand, they left dark brown stains on the skin. The light gray samples were slightly moist, loose, easily pulverized by hand, leaving a white residue. In total, 60 subsoil samples of approx. 1 kg each were collected from the 12 sites, from a depth of 10–20 cm.

The collected sewage sludge sample was tested for the following physical and chemical parameters: pH (PN-EN 12176:2004), moisture content (PN-EN 15934:2013-02E), total organic matter content (PN-EN 15935:2013-02E), sulfates (PN-ISO 9280-2002P), chlorides (PN-ISO 9297:1994P), phosphorus (PN-EN 14672:2006), and the total content of metals, including: Cd, Cr, Cu, Ni, Pb, and Zn (by digestion in a mixture of concentrated HCl + HNO_3_ at a 3:1 ratio, in a digester, at 130 °C). The collected subsoil samples (n = 60) were dried, sifted in a sieve with a particle size of < 1 mm, and subsequently tested for: pH (PN-EN 12176:2004), total organic matter content (PN-EN 15935:2013-02E), and total content of Cd, Cr, Cu, Ni, Pb, and Zn (by digestion in a mixture of concentrated HCl + HNO_3_ at a 3:1 ratio, in a digester, at 130 °C).

Element concentrations in the solution were determined in an accredited hydrogeochemical laboratory (certificate of accreditation: PCA AB1050) at the AGH University of Science and Technology. Cd, Cr, Cu, Ni, Pb, and Zn levels were determined with a precision of 10%, and an accuracy of 91%–108% (Table 1). The DL of the system used (Elan DRC-e ICP-MS system, Perkin Elmer) was 1 × 10^−5^ mg/dm^3^. The RTC-SQC001S, lot: LRA2028 (metals in sewage sludge) from Sigma-Aldrich was used as the reference material.

**Table 1:** Total content of selected metals in the certified reference material (CRM), and in the analyzed sewage sludge from the “Strole” sewage treatment plant and fecal values for the economic use of sewage sludge

Metal	CRM			Measured values^a^	Permissible content using^b^		
	Measured value	Certified value	AO (%)		In agriculture and land reclamation for agricultural purposes	For land reclamation for non-agricultural purposes	For the production of compost, for the cultivation of plants not intended for consumption and for the production of feed
	[mg/kg DM]						
Cd	359 ± 39	362 ± 37.8	99	6 ± 0.5	20	25	50
Cr	288 ± 33	317 ± 45.7	91	75 ± 0.5	500	1000	2500
Cu	651 ± 25	619 ± 18.7	105	220 ± 0.7	1000	1200	2000
Ni	488 ± 51	451 ± 45.7	108	17 ± 0.1	300	400	500
Pb	250 ± 29	256 ± 32.2	97	173 ± 1.1	750	1000	1500
Zn	755 ± 69	771 ± 63.9	98	1086 ± 3.3	2500	3500	5000

^a^Arithmetic mean with standard deviation for a series of three measurements in three replications

^b^According to Regulation of Environmental Ministry on municipal sewage sludge (2015)

*CRM* certified reference material

AO—analysis trueness (%)

The final stage of the study included an experiment involving the observation and evaluation of growth of a bulbous plant (bearded iris, *Iris barbata*) in two substrates: M1 (mixture of subsoil and sewage sludge) and M2 (subsoil only). The species was selected due to its popularity, relatively low subsoil requirements, high tolerance of heavy metals, and aesthetic qualities that may be significant for future use in park planning. 40 bulbs were planted in two alternating rows, at a distance of approx. 10 cm and a depth of 5 cm, 20 bulbs in each of the two substrates (series A and B). Vases with the seedlings were placed in a sunny spot so as to ensure adequate sunlight. The plants were watered every 2–3 days using 1.5 dm^3^ of tap water per vase. After approx. 3 months (87 days), the plants were gently separated from their substrate, rinsed with water, left to dry, and subsequently analyzed. The root section (five longest roots per bulb) and the oversubsoil part (leaf length and count) of each plant was measured.

As stated previously, the experiment was performed in two substrates. Substrate M1 was a mixture of sewage sludge and degraded subsoil at a 1:4 ratio (which means that 200 g of sewage sludge was found in 1 kg of the substrate M1). Substrate M2 only contained soil from the KZS “Solvay” brownfield land, averaged and homogenized from analogous portions collected from all 12 sampling sites.

All statistical analyses were performed using the Statistica v. 10 software.

## Results and Discussion

The analyzed sewage sludge had a neutral pH of 7.05. This potentially indicates that at the time of collection, the sludge was still undergoing methanogenic processes, though acid fermentation had already occurred. As reported by Szruba (2015), a pH value of 7.05 is consistent with poorly digested sludge.

The sludge had a very high natural moisture content, 81.59%, a total moisture content of 11.79%, and a hygroscopic moisture content of 13.37%. These parameters are particularly important in the context of potential incineration of sewage sludge. For comparison, the total moisture content of coal is 5.5%–8.6%, and its hygroscopic moisture content is 3.7%–4.6% (Kijo-Kleczkowska 2011; Wichliński et al. 2016). The values obtained for the analyzed sample are significantly higher, which can be a problem if thermal processing with energy recovery is considered, as a high water content significantly reduces the calorific value of a material.

The combustible matter content in the analyzed waste product was 72.40%, non-combustible − 27.60%. A high content of combustible components is favorable in the context of energy production, but may also indicate that the sludge is poorly digested (Szruba 2015). The volatile matter content was 71.82%, similar to that of straw (Kordylewski and Tatarek 2012), which is a satisfactory value for energy production. The organic matter content in the studied sewage sludge was 63.29%, and mineral content was 36.71%. These values indicate that the sludge was poorly stabilized. Typically, organic matter content is 60%–85% in fresh sludge, and ranges between 30% and 50% in well stabilized sludge (Przywara 2017). Organic matter content has a considerable impact on the calorific value of sludge, its soil forming properties, potential for biogas formation, and the problematic air odorization during land application of sludge (Podedworna and Umiejewska 2008). The obtained result is quite high, compared to that reported by Rosik-Dulweska et al. (2009), who found sewage sludge from the Zabrze treatment plant to have an organic matter content of 41.87%. This can be associated with the difference in area where the sludge was collected (urban vs. rural).

Humic substance content in the analyzed sample was approx. 2.09%, which is not high. Literature data indicate that any humic substance content lower than 3.5% should be considered relatively low (Kijo-Kleczkowska 2011; Wichliński et al. 2016). Sulfate content in the analyzed sewage sludge was approx. 8.1 g/kg (or 0.81 g/dm^3^ in water extract). Mean sulfate content in sewage from urban areas in Poland is 0.16 g/dm^3^ (Krzywy 1999), which means the present result was 5 times higher than the mean. The critical value of this parameter in sewage introduced into water or soil, defined in the applicable Regulation (2014), is 500 mg/dm^3^. As the sulfate content in water extract of the analyzed sludge was 0.81 g/dm^3^ (i.e. 810 mg/dm^3^), it warrants the conclusion that the analyzed sewage sludge exceeded the allowed sulfate content. However, considering the allowable sulfate content in mixing water used for concrete and other cement-based materials, which standard PN-EN 1008:2004 defines at 2 g/dm^3^, the obtained result is within this limit.

The chloride content in water extract of the analyzed sewage sludge was 23 mg/dm^3^. As the maximum content for sewage introduced into water or ground, defined by the applicable Regulation (2014), is 1000 mg/dm^3^, the chloride content in the analyzed sludge can be considered very low.

The phosphorus content in the analyzed sample was 150.7 mg/kg, or 0.015% of sample weight. According to literature data, mean phosphorus content in sewage sludge is 1.2%–2.7% (Krzywy 1999; Ociepa-Kubicka and Pachura 2013), while sludge from the “Czajka” treatment plant in Warsaw was found to have a phosphorus content of approx. 3.5%. Therefore, the content found in the present study can be considered low, and, surprisingly, is more similar to values found in sludge from meat processing wastewater (Rodziewicz et al. 2017). When analyzing the content of selected metals in the studied sewage sludge, the following mean values were found (in mg/kg dry weight): Cd 6, Cr 75, Cu 220, Ni 17, Pb 173, and Zn 1086 (Table 1). These results are rather similar to those reported by other authors, who also analyzed their material in the context of its application in land rehabilitation. In sewage sludge from the treatment plant in Busko-Siesławice, Gawdzik (2010) found a higher content of Cd (by 280%) and Pb (by 190%), and a lower content of Cr, Cu (by approximately 50%), Ni (by 90%) and Zn (by 20%).

Ociepa-Kubicka and Pachura (2013), who completed their research in the sewage sludge from the treatment plant in Dźbów, found a slightly higher content of Ni (by approximately 10%), but considerably lower content of Pb (by approximately 80%), Cd and Cr (by approximately 70%), Cu (by 30%), and Zn (by 10%). In a sample collected in Zabrze by Rosik-Dulewska et al. (2009), the researchers found 330% more Zn, 20% more Ni, and 10% more Pb. Lower values were found for Cu (by 30%), and Cd and Cr (by over 50%). The above differences are most likely due to differences in treatment plant location (urban vs. rural), as well as the different sewage treatment and sludge stabilization technologies.

A comparison of heavy metal content found in the sewage sludge from the “Stróże” treatment plant with the critical values for sludge used in non-agricultural land rehabilitation defined in the Regulation of the Minister of the Environment on municipal sewage sludge (2015) demonstrates that the studied sludge conforms with the regulatory requirements (Table 1). Moreover, the sludge can also be used in agriculture and in land rehabilitation for agricultural purposes, despite even stricter limits. The studied sewage sludge can also be freely used for compost production, non-food plant crops, and animal feed crops. It also contains significant amounts of sulfur (in the form of soluble sulfites), which can supplement its deficiencies in soils. Sulfur deficiency in the substrate reduces crop yields of crop plants, affecting the reduction of nitrogen uptake, reducing the content of sulfur-rich metabolites, which are responsible for plant resistance to biotic and abiotic stresses.

Subsoils in the brownfield land of the KZS “Solvay” plant had an alkaline pH ranging between 8.07 and 8.47, with a mean value of 8.30 (Table 2). Such strong subsoil alkalescence is naturally only found in very dry areas, and is very rare in Poland. In the studied area, this strongly alkaline pH was certainly caused by the calcium waste deposited there in the past.

**Table 2:** pH, total content of selected metals and TOC in subsoil degraded by the sodium processing industry

Parameter	pH	Cd	Cr	Cu	Ni	Pb	Zn	TOC
		[mg/kg DM]						[%]
Range	8.07–8.47	2.00–3.84	88.37–189.22	39.21–141.73	3.68–59.42	9.41–144.37	80.32–767.45	3.30–3.50
Av_A_	8.30	2.62	131.69	77.44	31.72	38.35	314.20	3.38
Av_G_	–	2.57	128.32	72.46	23.71	28.05	257.80	–
Me	–	2.54	125.81	73.12	30.10	26.28	268.07	–
SD	0.12	0.52	30.40	28.63	19.98	35.66	192.64	0.06
Natural content^a^	3.56–7.33	0.2–0.5	15–24	6–19	5–23	13–25	35–80	0.40–4.90
Class of contamination^b^	–	II	–	HI	11	I	II	–
Acceptable value^c^		2	200	200	150	200	500	–
Permitted value^d^	–	5 (0%)	200 (0%)	100 (17%)	60 (0%)	100 (8%)	300 (42%)	–

^a^According to Kabata-Pendias & Pendias (1999)

^b^According to TUNG guidelines for the assessment of soil pollution with heavy metals (Kabata-Pendias et al. 1993), for heavy type of soil, class: I—increased content, II—slightly pollution, III—medium pollution

^c^According to the Regulation of the Minister of the Environment on how to assess the pollution of the earths surface (2016), for I group of land (greenery areas, sports and recreation areas)

^d^According to the Regulation of the Minister of the Environment on municipal sewage sludge (2015), in bracket (X%) percentage of samples not meeting this requirement;

*Av*_*A*_ arithmetic average, Av_G_—geometric average, Me—median

“–”—no value

Humic substance content in the analyzed subsoil samples was 3.30%–3.50%, with a mean value of 3.38%, which should be considered high. This positively affects the sorption and buffering capacity of subsoil. Moreover, the presence of humic substances improves the overall structure of the subsoil, the hydrographic conditions, subsoil aeration, and subsoil microflora and microfauna activity. It also increases nutrient assimilation by plants, which is an important factor in land rehabilitation and ecological succession processes.

The total content of metals listed in the Regulation of the Minister of Environment (2015) was also determined in the studied subsoil samples. The content of Cd, Cr, Cu, Ni, Pb, and Zn fell within the following ranges (respectively, in mg/kg): 2.00–3.84, 88.37–189.22, 39.21–141.73, 3.68–59.42, 9.41–144.37, and 80.32–767.45. Compared to metal content naturally found in the topsoil in Poland, the present results were several times higher (Table 2): approximately: sixfold for Cd, 12-fold for Cu, fourfold for Ni, twofold for Pb, sevenfold for Zn, and nearly eightfold for Cr. In accordance with the soil contamination classification by the Institute of Soil Science and Plant Cultivation in Puławy, the measured Pb level falls within the “increased content” category (class I); Cd, Ni, and Zn levels fall within contamination class II, or “slight contamination”; while the Cu level is in contamination class III, or “moderate contamination”. In turn, in accordance with the Polish Regulation of the Minister of the Environment on the way of assessing the pollution of the earth’s surface (2016), with regard to class I land (marked in the zoning plan as cultivated green areas and/or sports and recreation areas), the allowed levels were only exceeded in the case of Cd and Zn, in 100% and 8% of the samples, respectively.

The limits defined by the Regulation of the Minister of the Environment on municipal sewage sludge (2015) were exceeded in the case of Cu, Pb, and Zn—in 17%, 8%, and 42% of the analyzed samples, respectively. However, due to the fact that only one statistical parameter (arithmetic mean) was used to calculate the mean metal concentrations, the potential use of the sewage sludge for land rehabilitation should not be precluded too hastily. For instance, in the case of Zn, where the allowed limit (i.e. 300 mg/kg) was exceeded in the largest number of samples, the highest variation also existed (SD = 192 mg/kg); the arithmetic mean for concentration in the studied subsoil was 314 mg/kg, but both the geometric mean (258 mg/kg) and the median (268 mg/kg) were well within the limit.

Therefore, when comparing metal content in degraded, heterogeneous subsoil with allowed regulatory limits, one should consider not only the arithmetic mean, but also the geometric mean, or the median, which is the most suitable measure for data sets with large variation (Table 2).

In the experiment, bearded iris (*I. barbata*) bulbs were planted in two substrates: M1 (a mixture of subsoil from the degraded area and sewage sludge at a 4:1 ratio), and M2 (a mixture of subsoil samples from the degraded area). As previously described, 20 bulbs were planted in each of the two substrates in two alternating rows (series A and B), at a distance of approx. 10 cm and a depth of 5 cm. After 87 days the plants were separated from the substrates, rinsed with water, and left to dry. Subsequently, the length of the root and oversubsoil parts of each plant were measured. The measurement included the length of 5 longest roots of each bulb, as well as the number and length of leaves. A macroscopic description of the plants was also developed.

**Fig. 2:**
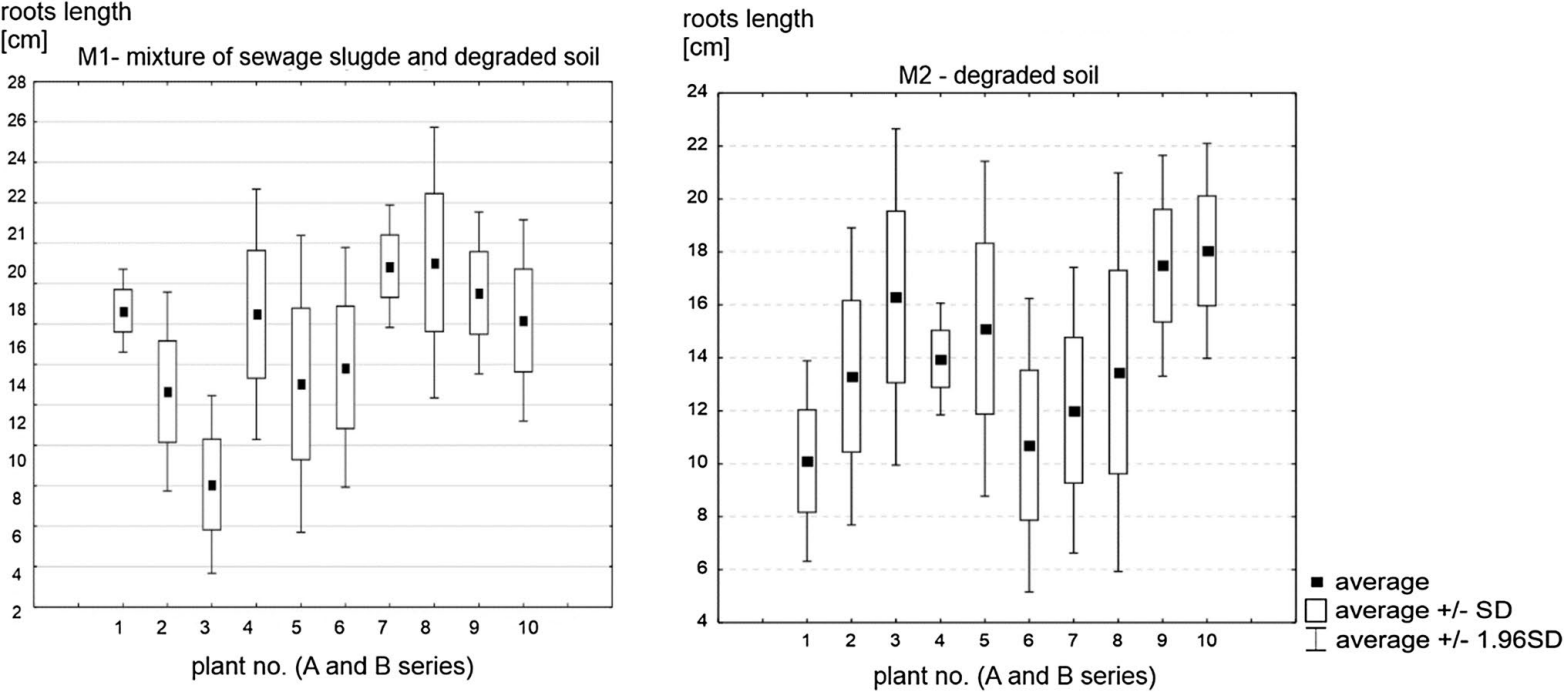
The length of the root system of plants growing on the M1 and M2 substrates

The roots of bulbs planted in substrate M1, a mixture of degraded subsoil and sewage sludge, were longer and denser than those of bulbs planted in degraded subsoil only, i.e. substrate M2. For all M1 bulbs, root length ranged between 8.1 and 19.0 cm. For M2, the range of root lengths was narrower, between 10.1 and 18.0 cm. The median root length of plants growing in substrate M2 was 13.7 cm, while that for substrate M1 was higher, 16.3 cm. In plants growing in substrate M1, the difference between the “weakest” and the “strongest” bulb was higher (Fig. 2). The differences were not, however, statistically significant for a threshold of *p* = 0.05. For bulbs planted in substrate M2, the growth patterns were more stable, with all bulbs retaining a nearly identical appearance throughout the vegetation period. This finding was likely due to the insufficient homogenization of the two ingredients of M1, causing some bulbs to have a locally better “access” to nutrients from the sewage sludge.

**Fig. 3:**
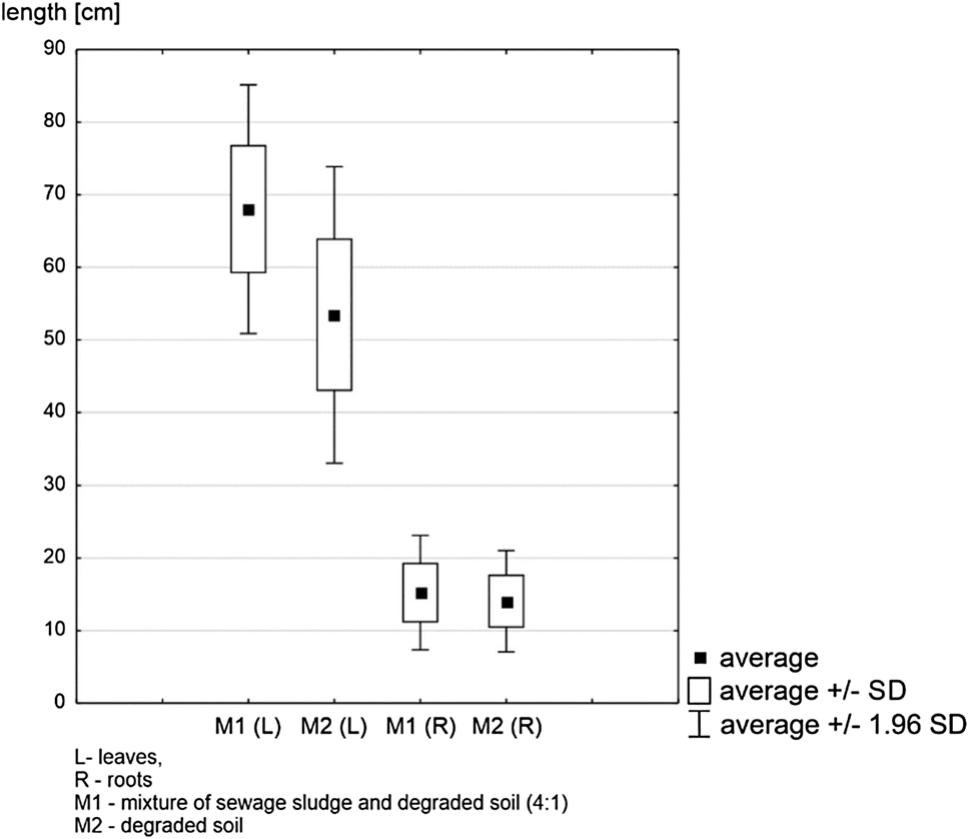
Average length of leaves and roots of all plants (n = 40) growing on substrates M1 and M2

For the oversubsoil parts, the observed tendencies were similar to those found in the root measurements. Plants growing in substrate M1 had visibly longer leaves than those planted in M2. Leaf lengths in substrate M1 ranged between 54.2 and 87.9 cm, while those in substrate M2 were significantly smaller, between 32.1 and 63.7 cm. Mean leaf length was 68.0 cm for M1, i.e. the mixture of degraded subsoil and sewage sludge, and 53.5 cm for M2, i.e. the subsoil degraded by the sodium processing industry (Fig. 3).

The differences were statistically significant (at *p* = 0.05). Therefore, the findings from the experiment indicate that sewage sludge from the “Stróże” treatment plant positively affected plant growth and improved the nutrient parameters of subsoil degraded by the KZS “Solvay” industrial plant.

Nonetheless, in future research, various doses of the sludge should be applied to the subsoil, in order to determine the optimum ratio of the mixture.

Sewage sludge from the “Stróże” treatment plant is within the metal content limits set by standards for land rehabilitation, both for non-agricultural and agricultural purposes. However, if the sludge were to be used for land rehabilitation, it should additionally undergo microbiological testing for *Salmonella* sp. bacteria, as well as for viable eggs of human and animal intestinal parasites, including *Ascaris* sp. *Trichuris* sp. and *Toxocara* sp.

The sludge has a relatively high sulfate content (8.1 g/kg dw), exceeding the regulatory limit for sewage introduced into water and subsoil. This is a potential environmental hazard in case of improper storage or possible drainage into subsoil and water, causing its degradation.

The sludge does have good parameters for thermal processing with energy recovery, i.e. a high content of combustible and volatile matter (72.40% and 73.40%, respectively), but due to its relatively high total (11.79%) and hygroscopic moisture content (13.37%), such use could offer no energy benefits. If it were to be used this way, improved dehydration, and additional, efficient drying processes would be required.

Ground in areas degraded by the KZS “Solvay” industrial plant has poor parameters in terms of pH (mean 8.3), and moderate parameters in terms of humic substance content (mean 3.38%). Therefore, the use of sewage sludge to improve the nutrient parameters of this subsoil for plant cultivation is indeed warranted.

An experiment using the bearded iris (*I. barbata*) demonstrated that sewage sludge from the “Stróże” treatment plant stimulates plant growth. Therefore, it can be used both for subsoil rehabilitation, and for fertilization. Still, more extensive research is required, including other plant species and a variety of sludge dosages. However, it is not recommended to use a single cumulative dose more than 30 Mg DM/ha per 2 years and 45 Mg DM/ha per 3 years.

Considering its properties and its broad spectrum of potential uses, sewage sludge should be viewed as a resource rather than a waste product. One must, however, bear in mind that in the case of land application of sewage sludge, the total content of heavy metals does not always reflect the actual risk of these metals’ infiltration into the environment. Leaching studies and testing for content of toxic substances such as PCBs, PAHs, PCDFs, PCDDs, and AOX are warranted in order to ensure maximum protection for the environment and human and animal health.
